# Perception of silent and motionless prey on vegetation by echolocation in the gleaning bat *Micronycteris microtis*

**DOI:** 10.1098/rspb.2012.2830

**Published:** 2013-03-07

**Authors:** Inga Geipel, Kirsten Jung, Elisabeth K. V. Kalko

**Affiliations:** 1 Institute of Experimental Ecology, University of Ulm, Albert-Einstein-Allee 11, 89069 Ulm, Germany; 2 Smithsonian Tropical Research Institute, Barro Colorado Island, Roosvelt Avenue, Tupper Building 401, Balboa, Ancón, Panamá, República de Panamá

**Keywords:** prey perception, echolocation, acoustic clutter, narrow-space active gleaning insectivorous bat, *Micronycteris microtis*

## Abstract

Gleaning insectivorous bats that forage by using echolocation within dense forest vegetation face the sensorial challenge of acoustic masking effects. Active perception of silent and motionless prey in acoustically cluttered environments by echolocation alone has thus been regarded impossible. The gleaning insectivorous bat *Micronycteris microtis* however, forages in dense understory vegetation and preys on insects, including dragonflies, which rest silent and motionless on vegetation. From behavioural experiments, we show that *M. microtis* uses echolocation as the sole sensorial modality for successful prey perception within a complex acoustic environment. All individuals performed a stereotypical three-dimensional hovering flight in front of prey items, while continuously emitting short, multi-harmonic, broadband echolocation calls. We observed a high precision in target localization which suggests that *M. microtis* perceives a detailed acoustic image of the prey based on shape, surface structure and material. Our experiments provide, to our knowledge, the first evidence that a gleaning bat uses echolocation alone for successful detection, classification and precise localization of silent and motionless prey in acoustic clutter. Overall, we conclude that the three-dimensional hovering flight of *M. microtis* in combination with a frequent emission of short, high-frequency echolocation calls is the key for active prey perception in acoustically highly cluttered environments.

## 1. Introduction

Many species of bats, toothed whales and even some birds [1–5] use biosonar to actively sense and perceive their environment. Biosonar permits orientation and is often also used for locating food under poor lighting conditions [6]. However, echolocation is generally thought to be of limited use for prey perception (including detection, classification and localization) in acoustically complex environments, such as dense understory vegetation. This is because the echolocator can experience auditory masking by the overlap of emitted signals and returning echoes (forward masking) and an overlap of target echoes with a multitude of overlapping echoes originating from the immediate surrounding, called acoustic clutter (backward masking) [7–9]. Effects of forward and backward masking leave species, such as bats, only a limited overlap-free window in which targets can be detected [7–9]. Masking effects can be a perceptual problem especially for bats hunting in narrow space such as forest understory, searching for insects close to vegetation and ground, or gleaning insects, fruit or nectar directly from the substrate [7–11].

To cope with the challenge of finding food in structurally complex environments, most bats use sensory cues provided by the food items and per definition forage in a passive mode [8]. These cues include olfactory [12] and visual ones [13,14], and prey-generated sounds [15–19], including rustling noises of walking or flying insects [17,20–24]. By contrast, active foragers in a narrow space rely on their own echolocation signals for prey perception [8]. They achieve a reduction of acoustic clutter by adjusting signal parameters such as bandwidth, duration, pulse interval and intensity during target approach [9,22,25–29]. However, these signal modifications can facilitate the perception of moving prey only close to, but not within, an echo-cluttered environment by enlarging the overlap-free window [26]. Given the complex sensorial task of foraging in complex acoustic environments, it is debated whether bats can extract enough information from echolocation alone for successful prey perception of silent and motionless prey within the clutter overlap zone ([8,9,15,22,23,30–32], but see [25,33]), and it has even been argued to be insurmountable [20].

The Neotropical common big-eared bat *Micronycteris microtis* (Phyllostomidae) is a small insectivorous gleaning bat (5–7 g) typically using small home ranges of 5.6 ha [34]. It forages within the vegetation of the dense rain forest understory, searching for prey on the vegetation by flying up and down single plants and briefly hovering in front of individual leaves (E. K. V. Kalko 1999, personal observation). *Micronycteris microtis* mainly preys on large insects, including beetles (Coleoptera), katydids (Orthoptera), caterpillars and moths (Lepidoptera), cicadas (Cicadina) [35] and occasionally small vertebrates [36]. Besides, more than 10 per cent of its diet consist of dragonflies (Anisoptera; [35]). Most of these large insects and especially the diurnally active dragonflies are caught while they are resting silent and motionless on the vegetation during night-time. This begs the question which sensory cues are used by *M. microtis* for successful prey perception?

In this paper, we investigate whether and how *M. microtis* is able to detect, classify and localize silent and motionless prey in acoustic clutter using echolocation. We predict that these species reveal specific strategies to reduce interference of clutter echoes on target echoes. Given its broad diet of silent and motionless prey items, we also predict that *M. microtis* uses echolocation as the sole sensory modality for prey detection in a highly cluttered environment. We further expected that the observed slow hovering flight along the vegetation is a species-specific behavioural strategy to classify and localize silent and motionless prey. In addition, we experimentally investigated the roles of prey characteristic cues (shape, surface structure and chitinous material) for detecting, classifying and localizing prey objects through echolocation on background vegetation. We hypothesize that a combination of prey characteristic cues is used by *M. microtis* for active prey perception and we propose that *M. microtis* uses an ‘acoustic search image’ to fulfil this task.

## 2. Material and methods

### (a) Study animals

**Figure 1.**
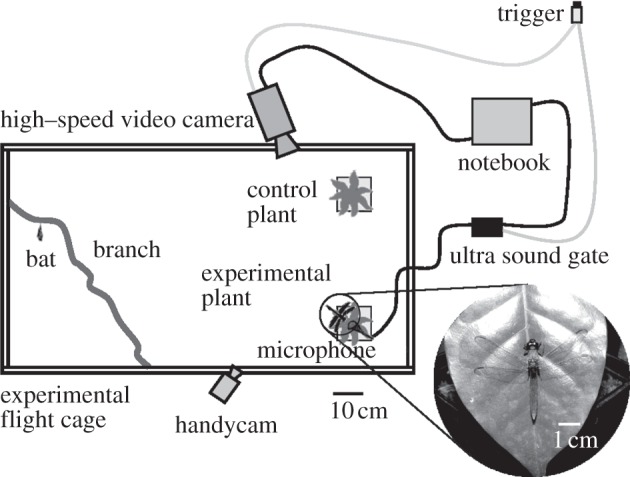
Experimental flight cage (1.40 × 1.00 × 0.80 m) viewed from the top with potted control and experimental plant. Target presentation on experimental plant (e.g. complete dragonfly).

We captured *M. microtis* (Phyllostomidae, Miller 1898; following the taxonomy of Simmons [37]), on Barro Colorado Island (BCI, 9°09″ N; 79°50″ W; Smithsonian Tropical Research Institute, STRI) and in Gamboa (9°07″ N; 79°41″ W) in Panamá. The experimentally naive individuals were transferred into a custom-made flight cage (1.4 0× 1.00 × 0.80 m; figure 1) located in the forest on BCI, which was exposed to the natural climate (70% humidity, 27°C) and sounds of the forest. All bats were allowed to adjust to this new environment for one night prior to the experiments. We conducted experiments with seven male bats for a maximum of two nights each (maximum 8 h per night). All individuals were released at the site of capture after completing the behavioural experiments. Except during experiments, water and food were provided ad libitum.

### (b) Experimental set-up

For our behavioural experiments, we placed two potted *Ormosia macrocalyx* (Fabaceae, height 22–30 cm) at one end of the flight cage, representing a control and an experimental plant (figure 1). A total of seven different targets (trials, see below and table 1) were presented on the experimental plant. Each trial started with presenting a target and ended after the bat's first reaction towards it. Subsequently, we presented another target while the target-position on the plant and the position of the experimental plant itself were randomly changed to minimize possible spatial learning. A high-speed camera (CamRecord 600, Optronis, Germany; 500 fps) was used to record the bats' behaviour in the vicinity of the experimental plant (visual field 30 × 50 cm) under infrared light conditions (wavelengths: 840–880 nm) beyond the phyllostomids' spectral range of vision [38]. Echolocation calls were simultaneously recorded (using a 5 s post trigger) with a condenser microphone (microphone capsule CM16, CMPA preamplifier unit, Avisoft Bioacoustics, Berlin, Germany) and digitized using a real time ultrasound acquisition board (UltraSoundGate 116, Avisoft Bioacoustics, Germany; 500 kHz sampling rate, 16 bit resolution). In addition, the bats' behaviour in the flight cage was continuously monitored with an infrared-sensitive camcorder (DCR-H C39E, SONY, Japan).

**Table 1.** Seven presented targets providing differences in shape, surface structure and material. (Each target was presented only once to each individual. The target order was randomized for each individual.)

target	shape	surface structure	material
(i) complete dragonfly	cross-shape	rough	chitin
(ii) four wings	linear	rough	chitin
(iii) two wings	linear	rough	chitin
(iv) dragonfly body	linear	smooth	chitin
(v) smooth-winged aluminium dummy	cross-shape	smooth	aluminium
(vi) crumpled-winged aluminium dummy	cross-shape	rough	aluminium
(vii) paper dummy	cross-shape	smooth	paper

### (c) Presented targets

We tested the importance of prey-specific cues such as shape, surface structure, and material, for detection, classification and localization by presenting seven different targets to the bats (table 1). To investigate the importance of prey shape, we offered a (i) complete dragonfly, which is characterized by a cross-shape of the dragonfly body and its wings, raised wing venation and chitinous material. Additionally, the cross-shape was altered by presenting (ii) four or (iii) two dragonfly wings without the body or (iv) the dragonfly body without any wings. Dragonfly dummies made from aluminium with either (v) smooth or (vi) crumpled wings were used to test for the importance of surface structure. Finally, we presented (vii) paper dragonfly dummies to investigate the effects of the chitinous material on prey perception. All dummies imitated shape and size of natural dragonflies.

### (d) Analysis of flight and echolocation behaviour during target approach

The recorded bats' flight and echolocation behaviour during target approach was synchronized using Highsync v. 0.94 (Slomotec, Germany). Subsequently, we compared the duration of the approach flights towards the seven different target conditions using a repeated-measures ANOVA (Statistica, v. 6.1, StatSoft, Oklahoma, USA). To assess which of the seven targets were classified as potential prey, we counted the number of first reactions towards the targets. Attacking or landing was scored as positive reaction; hovering in front of the target without an attack was noted as rejection. For statistical analysis of positive reactions and rejections, we applied a Pearson's *χ*^2^-test with a randomization procedure, based on 2000 randomizations (*p*-value for low sample size, [39]) using the statistics program R (v. 2.6.0, The R Foundation for Statistical Computing). Cramer's *V*-test, a measure of association based on *χ*^2^ that reports a value for the association between two variables, was used to support our results from the randomizations.

Precision of prey localization was analysed from the high-speed video recordings (software CamControl v. 1.23, Optronis, Germany). We discriminated between targets with body (i, iv–vii) and targets without body (ii, iii). We then assessed which body parts were attacked by the bats (thorax/head, centre between wings/wings).

Echolocation calls emitted during target approach were analysed using Avisoft SASLabPro v. 4.40 (Avisoft Bioacoustics, Berlin, Germany). Each sequence was high-pass filtered (Tschebyscheff, 20 kHz). Spectrograms were generated using a Hamming window (512 fast Fourier transform, 96.87% overlap) resulting in a frequency resolution of 977 Hz and a time resolution of 0.032 ms. Echolocation call parameters such as start, end and peak frequency (i.e. frequency with the highest amplitude) were taken from the spectrograms. In addition, we calculated call duration (ms), inter and intra call groups pulse interval (ms), total bandwidth (kHz), duty cycle (%), sweep rate (kHz/ms) and repetition rate (Hz). We pooled sequences per individual (*n* = 7) and calculated mean and standard deviation (s.d.) of each parameter (means of means).

## 3. Results

### (a) Overall search flight behaviour and prey detection

In all experimental trials, *M. microtis* flew towards the control and the experimental plants and briefly hovered in front of and along each plant inspecting individual leafs for the presence of any target of interest. When detecting a target, the bats directed head, ears and noseleaf towards the potential prey item and started hovering in front of it. All individuals continuously emitted echolocation calls, ensonifying the potential target of interest from different angles (hereafter: scanning). This foraging behaviour is similar to our observations in nature ([34], E. K. V. Kalko 1999, personal observation). All seven presented targets were detected and scanned by all individuals and none of them were ignored. Scanning was never observed in front of leaves without a target.

### (b) Flight behaviour during target approach

**Figure 2.**
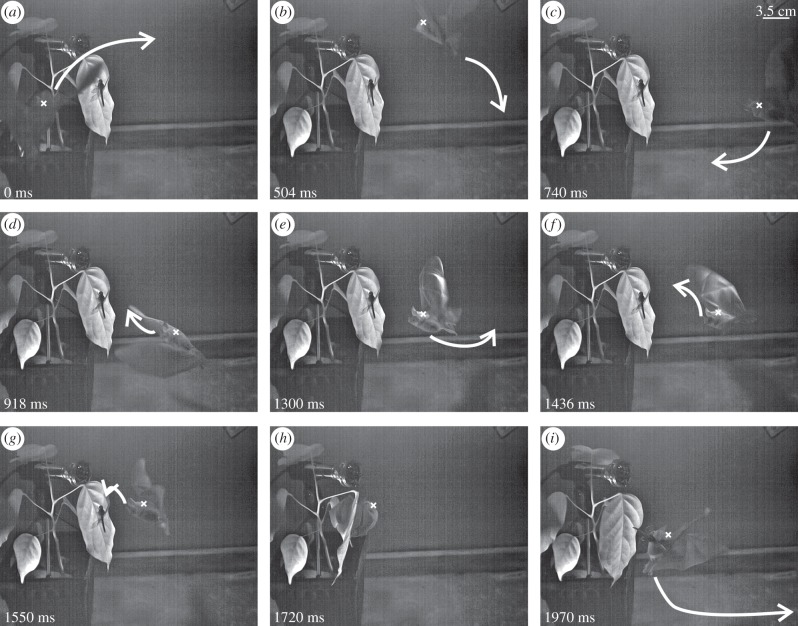
Picture series gained from a high-speed infrared video recording (EMS) of *M. microtis* scanning and acquiring target (complete dragonfly): white arrows indicate the subsequent direction of the individual's movement. Small white cross marks the back of the bat's head. (*a*) Beginning of the scanning behaviour with the bat moving upwards, to the right-hand side of the leaf. (*b*) Movement from the right-hand side (distance to prey *ca* 10 cm) of the leaf downwards. (*c*) Movement towards the centre of the leaf with the dragonfly (distance *ca* 14 cm). (*d*) The bat flies closer towards the leaf while moving slightly upwards (from a distance of *ca* 8 cm to a distance of *ca* 5 cm to prey). Bat hovering on the spot close to prey. (*e*) With the head directed towards the prey, the bat briefly flies backwards (approx. 5 cm). (*f*) Bat changes flight direction again and moves forward with its head turned slightly upwards (distance approx. 9 cm). (*g*) Final approach. (*h*) End of scanning by touching the experimental leaf and landing on the prey. Taking prey off the leaf. (*i*) Take off with the dragonfly.

During target approach, *M. microtis* showed a stereotypic flight behaviour (hereafter: scanning behaviour) characterized by flight movements covering a three-dimensional space within a radius of less than 15 cm in front and around the potential target (see figure 2 and electronic supplementary material, video). In general, *M. microtis* approached the potential target of interest in a horizontal flight path from one side (figure 2*a*), moving subsequently in a diagonal line slightly upwards across the leaf to the other side (figure 2*b*), always facing the potential target. This movement was in some cases repeated two or three times. Individuals then hovered downward to the centre of the leaf (figure 2*c*) approaching the target to about 8 cm (figure 2*d*). Here, individuals hovered briefly, sometimes slightly moving up and down in front of the target (figure 2*e*), approaching it to about 5 cm. Individuals subsequently moved backwards again to a distance of approximately 9 cm (figure 2*f*). Then they abruptly approached the target from a slightly higher position (figure 2*f,g*) and landed on it (figure 2*h*). Bats then took the target and left (figure 2*i*) to their preferred perch in the flight cage. In case of target rejections, the scanning behaviour ended after close hovering flight (figure 2*e*), as was indicated by a shift and turn of the head, ears and noseleaf away from the experimental leaf.

### (c) Echolocation behaviour

During hovering flights bats continuously emitted broadband (83.4 ± 9 kHz total bandwidth) frequency-modulated (FM), multi-harmonic echolocation calls, ranging from 143.3 ± 18 kHz (start frequency) to 68.6 ± 9 kHz (end frequency). Echolocation calls were of very short call duration (0.2 ± 0 ms) and emitted either as single pulses with an inter-pulse interval of 30.6 ± 2 ms or in groups of two calls with an intragroup pulse interval of 14.5 ± 1 ms. Only shortly before landing longer groups containing three to four signals were emitted. During final target approach, none of the individuals showed a terminal phase. Echolocation calls were emitted at a consistent repetition rate of 48.1 ± 6 Hz at a duty cycle of 0.9 per cent for sound emission. The power spectrum of the echolocation signals usually showed three frequency peaks, which roughly reflected the best frequencies (frequencies with maximum amplitude) of three harmonics; a second harmonic at 68.2 ± 3 kHz, a third harmonic at 98.5 ± 2 kHz and a fourth harmonic at 127.9 ± 2 kHz, while the fundamental frequency was typically too suppressed to be accurately measured. Echolocation parameters across all individuals are presented in table 2.

**Table 2.** Echolocation call parameters of seven *M. microtis* during scanning behaviour. (The mean of each parameter was calculated based on the mean of four to seven sequences for each individual (42 sequences in total, 1792 echolocation calls).)

call parameter		mean ± s.d.
pulse duration (ms)		0.19 ± 0
start frequency (kHz)		143.30 ± 17.5
end frequency (kHz)		68.57 ± 9.1
bandwidth (kHz)		83.38 ± 8.9
pulse interval (ms)	intragroup	14.49 ± 0.8
	intergroup	30.61 ± 2.2
peak frequency (kHz)	peak frequency 1	68.19 ± 2.9
	peak frequency 2	98.51 ± 2.3
	peak frequency 3	127.89 ± 1.6
duty cycle (%)		0.93 ± 0.1
sweep rate (kHz/ms)		459.83 ± 77.9
repetition rate (Hz)		48.14 ± 5.6

### (d) Differentiation of targets

**Figure 3.**
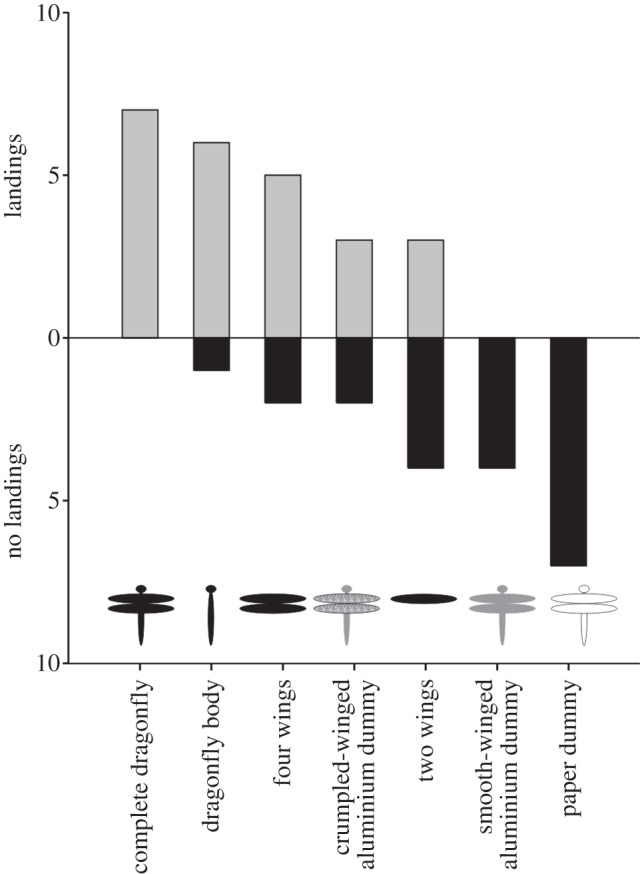
Number of landings on the seven presented targets versus prey rejections of seven *M. microtis* individuals.

*Micronycteris microtis* did discriminate between the seven different targets (*χ*² = 23.027, *p* < 0.001; *n* = 44) and we found a significant relationship between the number of landings and the respective target (Cramer's *V* = 0.723; figure 3). While all individuals classified complete dragonflies as potential prey items and collected them from the leaf, scanning behaviour was always aborted if the bats encountered the paper dragonfly dummy or the aluminium dummy with smooth wings. All other targets, the dragonfly body without wings, the four and two wings without body and the aluminium dummy with crumpled wings (in order of decreasing attractiveness) did provoke some individuals to continue scanning and ended with the collection of the targets (figure 3).

Ninety-eight per cent of the recorded scanning behaviours lasted less than 3 s (mean 1.27 ± 0.78 s, *n* = 44) and we observed no significant differences in the scanning time between the seven different targets (ANOVA: *F*_6,37_ = 0.77, *p* = 0.6). This indicates that *M. microtis* needs less than 3 s to classify targets of interest as potential prey (or not) within a dense and acoustically cluttered environment. While this is a rather short period of time, it is far longer than in aerial hawking bats that detect and attack airborne targets in uncluttered space (see [9] for review).

### (e) Precision of target localization

In all instances of successful target collection, we observed a high precision of prey localization. In 93 per cent of target collections where the body of a dragonfly was part of the presented target, *M. microtis* bit into the thorax right at the base of the wings. Only once the bite was directed to the head (7%). If no dragonfly body was part of the presented target, the bats bit into the wings close to their base (80%) or right into the place where the thorax would have been in an intact dragonfly (20%).

## 4. Discussion

Owing to acoustic masking [8,9], active perception of silent and motionless prey in dense understory vegetation by echolocation alone has long been regarded impossible [8,9,20]. As predicted, here we present, to our knowledge, the first experimental evidence that the gleaning bat *M. microtis*, using FM calls, is able to detect, classify and precisely localize silent and motionless prey within the clutter overlap zone by echolocation alone using a species-specific strategy. The ability to detect prey in acoustic clutter by echolocation alone has been highly debated, and it has been argued that bats gleaning insects from the vegetation need prey-specific cues such as vision, olfaction or prey-generated sounds for prey detection and localization [9,20,40,41] or if hunting actively using echolocation alone exhibited little tolerance of overlap between prey and clutter echoes [26].

During target approach, *M. microtis* displayed a stereotypical three-dimensional hovering flight which started as soon as an individual detected a target. We refer to this behaviour as scanning behaviour because it involved an ‘ensonification’ of the potential target from different angles with echolocation calls right up until landing. Considering *M. microtis*' signal duration, targets must have been at least 3.3 cm in front of any background to prevent backward masking [42]. Owing to the minute distance between the dragonfly wings to the background leaf (approx. 0.5 cm), *M. microtis* should face backward masking effects. However, here we argue that through the three-dimensional flight behaviour, *M. microtis* alters the relative angle between target and background to reduce clutter interferences and thus the backward masking effect. This is supported by previous publications which showed, that on smooth surfaces prey echoes appear stronger in contrast to the weaker background echoes because parts of the background echoes are reflected away from the echolocator (mirror effect; [43]). We propose that *M. microtis* uses a similar strategy for target detection, classification and localization, just in the vertical plane along the vegetation (I. Geipel 2010, personal observation).

As predicted, *M. microtis* used echolocation as the sole sensory modality for prey perception in the highly cluttered space of understory vegetation. In general, narrow-space foragers hunting in dense echo-cluttered environments have rather short, broadband echolocation signals, emitted at very short pulse intervals [9]. Short call durations are considered as an adaptation to reduce forward masking effects [9]. In *M. microtis*, a call duration of 0.19 ms results in a minimum detection distance without forward masking of 3.3 cm ((pulse duration × 348.8 m s^−1^)/2; [42]). However, during scanning, *M. microtis* usually kept a distance of 5–15 cm towards the targets and thus we argue that this species actively avoided forward masking.

Echolocation signals of *M. microtis* showed a broad bandwidth in a very high-frequency range. Broad bandwidths of echolocation signals allow high target resolution in the spectral domain [44,45]. Depending on the emitted frequency, differences in depth can cause distinct, sharp absorption peaks in the frequency spectrum of echoes due to interference patterns and can be discriminated down to 1 mm [46]. We thus argue that signal design in *M. microtis* is the perceptual basis for target recognition and discrimination, as it very likely provides prey characteristic echoes with patterns or notches in the spectral domain [47]. Similar to other narrow-space foragers, *M. microtis* emitted echolocation signals at very short pulse intervals to provide a continuous flow of information during hovering flights and target approach. However, a terminal phase just before prey attack was never observed. The absence of a terminal phase is known from most gleaning bats [12,13,21,22,48,49]. This supports the hypothesis that a terminal phase seems to be only of particular importance for tracking moving prey [50]. Just recently it has been proposed that prey perception by *M. microtis* could be facilitated by air turbulences produced by bat wing air forces during hovering flights through slight, induced vibrations of prey body parts [51]. Although we cannot exclude this possibility, from our results this seems unlikely, because *M. microtis* readily detected prey items lacking potentially vibrating body parts such as wings. We rather argue that the observed three-dimensional hovering flight in combination with constant emission of short, high-frequency echolocation calls is the species-specific strategy of *M. microtis* and the key for prey perception in an acoustically highly cluttered environment.

As predicted, *M. microtis* is able to classify different targets based on shape, surface structure and material using echolocation. Because all individuals clearly preferred four over two wings, we suggest that the characteristic cross-shape of a dragonfly is important for prey classification. Dragonfly bodies lacking wings were possibly classified as another type of prey that is part of *M. microtis*' natural diet (e.g. caterpillars, stick insects (Phasmatodea), [35]) owing to elongated shape. As bats scanned but rejected smooth-surfaced targets and took strongly structured surfaced dummies, we infer that differences in reflective properties of surface textures are recognized by *M. microtis* and used for prey classification. The importance of texture information for bats has been already demonstrated for the Indian false vampire bat, *Megaderma lyra* (Megadermatidae) [52]. Additionally, different target material might vary in spectral properties and echo strength owing to sound reflectivity. Hence, *M. microtis* may not have perceived relatively weak echoes of paper dummies as characteristic prey echoes, while it classified stronger echoes of the aluminium dummies and the chitinous dragonfly body without wings as prey items. The ability to discriminate between different materials is further supported by the observation of *M. microtis* detecting and gleaning motionless stick insects in the dark (I. Geipel 2006, personal observation) and the regular occurrence of phasmids in its diet [35]. Given the cluttered situation in the forest, *M. microtis* must be able to distinguish between a chitinous stick insect and, for instance, a small twig.

Nevertheless, our results indicate that the cross-shape, surface structure of the wings, and material of dragonflies cannot be regarded independently as factors eliciting capture attempts. Instead, they have to be regarded as a suite of traits that together allow a successful classification and localization in clutter. We thus propose that *M. microtis* perceives an acoustic image of a target based on the combination of several acoustic target characteristics. This coincides with Schmidt [33] suggesting that gleaning bats using echolocation form an acoustic image based on shape and structure of an object, and Simmons *et al.* [53] who discussed in detail how FM-bats obtain acoustic images from temporal and spectral echo information. In addition, we suggest that a received acoustic image might be compared with an innate template. This might explain the capture success of 86 per cent for the body without wings, where the cross-shape and the wings' surface structure were missing, but which resembled other prey items of the natural diet in shape and material. The observed very high precision in target localization that even extended to targets missing characteristic body parts, suggests that *M. microtis* must receive a very precise acoustic image from target echoes and may be able to extrapolate the presumed overall shape based on a pre-existing template. An acoustic image of different prey types might even be learned in infants as in *M. microtis* weaned subadults are fed by their mothers with solid prey items over a period of several moths [54]. However, we like to state, that to further elucidate aspects of an acoustic image by the reflective characteristics of prey items, it will be necessary to conduct ensonification experiments with prey in different background situations.

In summary, we here present, to our knowledge, the first experimental evidence that the untrained gleaning bat *M. microtis* uses echolocation alone for a successful detection, classification, and precise localization of silent and motionless prey in a highly cluttered environment. Until now, it has been largely debated if this is possible without using additional cues (vision, olfaction and acoustic) for prey perception [20]. Here we suggest that the stereotypical three-dimensional hovering flight, while constantly emitting echolocation calls, as a species-specific behavioural strategy is the key for prey perception in a highly cluttered environment. From our results, we further conclude that *M. microtis* perceives a detailed acoustic image of prey shape and structure through its echolocation calls. We thus infer that through these adaptations, *M. microtis* gains access to resources unavailable to other gleaning bats and we propose adding *M. microtis* to the category ‘narrow space active gleaning insectivores’ of the ecological guild system [8,9].
